# The red pepper’s spicy ingredient capsaicin activates AMPK in HepG2 cells through CaMKKβ

**DOI:** 10.1371/journal.pone.0211420

**Published:** 2019-01-29

**Authors:** Alicia Bort, Belén G. Sánchez, Elena Spínola, Pedro A. Mateos-Gómez, Nieves Rodríguez-Henche, Inés Díaz-Laviada

**Affiliations:** 1 Department of Systems Biology, School of Medicine and Health Sciences, University of Alcala, Alcalá de Henares, Madrid, Spain; 2 University Institute of Biomedical and Health Research (IUIBS), Department of Biochemistry and Molecular Biology, Physiology, Genetics and Immunology, University of Las Palmas de Gran Canaria, Las Palmas, Spain; 3 Chemical Research Institute “Andrés M. del Río” (IQAR), University of Alcalá de Henares, Madrid, Spain; Univerzitet u Beogradu, SERBIA

## Abstract

Capsaicin is a natural compound present in chili and red peppers and the responsible of their spicy flavor. It has recently provoked interest because of its antitumoral effects in many cell types although its action mechanism is not clearly understood. As metabolic dysregulation is one of the hallmarks of cancer cells and the key metabolic sensor in the AMP-activated kinase (AMPK), in this study we explored the ability of capsaicin to modulate AMPK activity. We found that capsaicin activated AMPK in HepG2 cells by increasing AMPK phosphorylation and its downstream target ACC. Mechanistically, we determined that capsaicin activated AMPK through the calcium/calmodulin-dependent protein kinase kinase β, CaMKKβ as either the CaMKK inhibitor STO-609 or CaMKK knock down with siRNA abrogated the activation of AMPK. Moreover, capsaicin decreased cell viability, inhibited Akt/mTOR pathway and increased reactive oxygen species (ROS) in HepG2 cells. AMPK activation was involved in the underpinning mechanism of capsaicin-induced cell death.

## Introduction

Natural compounds and dietary products provide an interesting area of research because of their low toxicity and potent efficacy. Capsaicin (CAP) is a natural alkaloid and the main active ingredient of spicy peppers belonging to *Capsicum* genus. It is used as additive in food in many cultural cuisines and it is responsible for the hot or burning sensation experienced on contact with chili peppers. Although traditionally associated with analgesic effects, it has been recently proposed that capsaicin also displays antitumor activity in various cell types and enhances the sensitivity of cancer cells to cytotoxic drugs [1–3]. In addition, laboratory data support the notion that capsaicin could act as an anti-obesity drug by increasing energy expenditure [4–6]. It has recently been shown that the intake of capsaicin reduces the insulin resistance caused by obesity in rats [7, 8]. Moreover, epidemiological data reveal that consumption of foods containing capsaicin is associated with a lower prevalence of obesity [9, 10]. Cancer cells undergo a metabolic reprogramming in order to satisfy energy demands of a continuous growth. Even in the presence of oxygen, tumors maintain anaerobic glycolysis to ensure enough levels of carbohydrate intermediates for anabolic reactions, as described by Otto Warburg nine decades ago [11]. Furthermore, recent research indicates that metabolites themselves can be oncogenic by altering cell signaling and blocking cellular differentiation [12]. Therefore, to impact metabolic reactions in cancer cells may be a new therapeutic strategy for this disease.

Hepatocellular carcinoma (HCC) remains one of the most common and lethal malignancies worldwide despite the development of various therapeutic strategies. The prognosis for patients with advanced HCC remains extremely poor due to the high rates of recurrence and metastasis. The liver is the major metabolic organ and dysregulation of metabolic balance has been reported to cause liver diseases including cancer [13].

The key metabolic sensor for the cell energy status is the enzyme AMP-activated kinase (AMPK). Its activation leads to the implementation of catabolic pathways in order to restore ATP levels. Activation of AMPK is regulated by phosphorylation and allosteric modulation. Phosphorylation at the conserved residue of Thr172 in the catalytic domain increases about 500-fold AMPK activity. The main upstream kinases that phosphorylate AMPK are liver kinase B1 (LKB1) and the kinase that phosphorylates Ca^2+^/calmodulin dependent kinase type β, (CaMKKβ, also known as CaMKK2) [14]. In addition, AMP exerts an allosteric activation by increasing the AMPK activity by 5-fold [15].

The importance of AMPK as a therapeutic target in cancer is beginning to be unveiled. Clinical data suggest a greater benefit of anticancer therapy in patients with type 2 diabetes mellitus treated with metformin, an activator of AMPK. [16]. It has also been recently observed that AMPK may be involved in the appearance of resistant phenotypes. For example, the loss of LKB1 in breast cancer cells increases the aggressiveness, migration ability and appearance of stem-like phenotype whereas the activation of LKB1, and consequently of AMPK, reduces the formation of mamospheres and the expression of pluripotent factors [17].

Therefore, AMPK is emerging as a new therapeutic target which, through metabolism and signaling regulation, could reduce cancer progression.

In the present study we examined whether CAP could modulate AMPK in HCC HepG2 cells. We found that CAP increased phosphorylation of AMPK and its downstream effector ACC through a mechanism involving CaMKK and calcium. AMPK activation by capsaicin is involved in the underlying mechanism of capsaicin-induced cell death.

## Materials and methods

### Reagents

Capsaicin and capsazepine (CPZ) were obtained from Tocris (Ellisville, USA). STO-609 and BAPTA were obtained from Sigma (St. Louis, MO, USA). The AMPK inhibitor Dorsomorphin was obtained from Tocris Bioscience (Bristol, UK).

### Cell culture

The human hepatocellular carcinoma HepG2 cells line was purchased from the American Type Culture Collection (ATCC HB-8065, Rockville, MD, USA) and incubated at 37 °C in a humidified atmosphere with 5% CO2 and cultured in DMEM/10%FBS supplemented with 1% non-essential amino acids and 100 IU/mL penicillin G sodium, 100 μg/mL streptomycin sulfate, 0.25 μg/mL amphotericin B (Invitrogen, Paisley, UK).

### Cell viability assay

Viability of HepG2 cells was assessed by the MTT assay. Cells were seeded at a density of 5x10^3^ cells/well on 12-well plates and when attached and grown treated with capsaicin according to the experiment. Then, 200 μL of MTT solution (Sigma, St. Louis, MO, USA) was added to each well and incubated at 37°C for 4 h. The supernatants were then replaced with 2-propanol and incubated for complete solubility of formazan crystal. The optical density of each well was then determined at 570 nm using a microplate reader (iMARK, BioRad Laboratories, Inc, Hercules, CA, USA). Each experiment was performed in triplicate. Cell viability was calculated as a percentage compared to the control cells, which were arbitrarily assigned 100% viability.

Cell viability was also determined by counting viable and dead cells by Trypan blue staining. Trypan blue positive and negative cells were counted using a Countess automated cell counter (Invitrogen, Carlsbad, CA, USA). Results were expressed in relation to total number of cells counted.

### Flow cytometry

Flow cytometry was used to detect the distribution of cell cycle and apoptotic cells. After being cultivated with medium alone or medium containing the indicated stimuli, 10^5^ cells in 35 mm culture dish were harvested in 0.35% trypsin, collected and fixed with 70% cold ethanol at 4°C for 1h. Then, cells were centrifuged at 1500g for 5 min and incubated in 0.5 ml PBS containing 0.1 mg/ml RNase for 30 min at 37°C. DNA staining was performed adding 5 μl propidium iodide (Invitrogen, Eugene, Oregon, USA). Apoptosis was evaluated at 24 h following treatment using an Annexin V-fluorescein isothiocyanate (FITC) Apoptosis Detection kit according to the manufacturer’s instructions (BD Biosciences, San Diego, CA USA). Briefly, cells were washed twice with PBS and digested with 0.25% trypsin for 5 min. Cell were then centrifuged at 1500 g for 5 min and incubated in 0.5 ml of Binding Buffer (10 mM HEPES pH 7.4, 150 mM NaCl, 2.5 mM CaCl2, 1 mM MgCl2, and 4% BSA), with 4 μg/ml Annexin V-FITC for 15 min. Cells were then washed in PBS and resuspended in Binding Buffer with 0.6 μg/ml Propidium Iodide (PI). Data acquisition and analysis were performed in a FACSCalibur flow cytometry system (BD Biosciences, San Jose, CA, USA) using Cyflogic software V1.2.1 (Perttu Terho, Mika Korkeamaki, CyFlo Ltd, Turku, FINLAND). A total of 5×10^4^ events were collected for each sample.

### Measurement of ROS

The intracellular level of ROS were analyzed with the oxidation-sensitive fluorescent probe 2′,7′-Dichlorofluorescin diacetate (DCFDA), (Sigma, St. Louis, USA). After treatments, cells were incubated with 5 μM DCFDA for 20 minutes in a 37°C incubator. Fluorescence was measured by a FACSCalibur flow cytometer. The fluorescence corresponding to the oxidized probe was followed by measuring the green (530 mm; FL1) fluorescence in the iodide propidium negative population (585 mm; FL2). Untreated cells without fluorescence were used as the background fluorescence.

### Fluorescence microscopy

Morphological changes in the nuclear chromatin of cells undergoing apoptosis were detected by staining with the DNA binding fluorochrome 4′,6-diamidino-2-phenylindole (DAPI). Cells were grown in glass coverslips and after treatment, coverslips were incubated with a drop of 1 μg/ml DAPI for 20 min. Coverslips were then washed in PBS, mounted with Vectashield (Vector Laboratories, Burlingame, CA, USA) and observed by fluorescence microscopy.

### Western blot analysis

After treatments or transfection for 48 h, cells were harvested and proteins were extracted using lysis buffer (50 mM Tris pH 7.4, 0.8 M NaCl, 5 mM MgCl_2_, 0.1% Triton X-100) containing Protease Inhibitor and Phosphatase Inhibitor Cocktail (Roche, Diagnostics; Mannheim, Germany), incubated on ice for 15 min and cleared by microcentrifugation. Protein concentrations were measured by BioRad protein assay kit (Richmond, CA, USA. Equal amounts of protein (20 μg) were loaded in each lane with loading buffer containing 0.125 M Tris-HCl (pH 6.8), pH 6.8, 20% glycerol, 4% SDS, 10% mercaptoethanol and 0.002% bromophenol blue. Samples were boiled for 5 min before being separated on 8–15% SDS-PAGE gels, depending on protein to be analyzed. After electrophoresis, proteins were transferred to polyvinylidene difluoride membranes (BioRad) using an electrophoretic transfer system (Bio-Rad, Hercules, CA, USA). The membranes were then incubated overnight at 4 °C with one of the following the specific primary antibodies: anti-p-AMPKα1-thr172, anti-AMPK, anti-p-ACC-ser79, anti-ACC, anti-p-LKB1-ser428, anti-LKB1, anti-CaMKKβ, anti-TRPV1 (Cell Signaling Technology, Danvers, MA, USA). After being washed, Horseradish peroxidase-linked goat anti-mouse and goat anti-rabbit IgG secondary antibodies were then added at a dilution ratio of 1:2000 and the membranes were incubated at room temperature for 2 h. The immune complex was visualized with an ECL system (Cell Signaling Technology, Danvers, MA, USA).

### siRNA transfections

Cells were transfected in 1 ml OPTIMEN containing 4 μg lipofectamine iMax (Invitrogen, Carlsbad, CA), with 100 nM AMPK specific small interfering RNA (siRNA) duplexes (5'-CCCAUAUUAUUUGCGUGUAdTdT-3' and 5'-UACACGCCAAAUAAUAUGGGdTdT-3') (Ambion-Life Technologies, Carlsbad, CA, USA), 100 nM CaMKKβ selective si RNA (5'-GCUCCUAUGGUGUCGUCAAdTdT-3' and 5'-UUGACGACACCAUAGGAGCdTdT-3') or control scrambled RNA according to manufacturer’s protocols (Invitrogen, Carlsbad, CA). At 48 h after transfection, the medium was removed and replaced for DMEM. At dedicated time points after transfection, cells were used for MTT cell viability assays or Western blot.

### Statistical analysis

All values are expressed as the mean ± SEM. Significances of the differences were assessed by student’s t test using GraphPad 6.0 (La Jolla, CA, USA) software. Numbers of replicates (n) refer to biological replicates, i.e. the number of independent cell cultures analyzed. Statistical significance was defined as p < 0.05 for all analyses.

## Results

### Capsaicin activates AMPK in HepG2 cells

**Fig 1:**
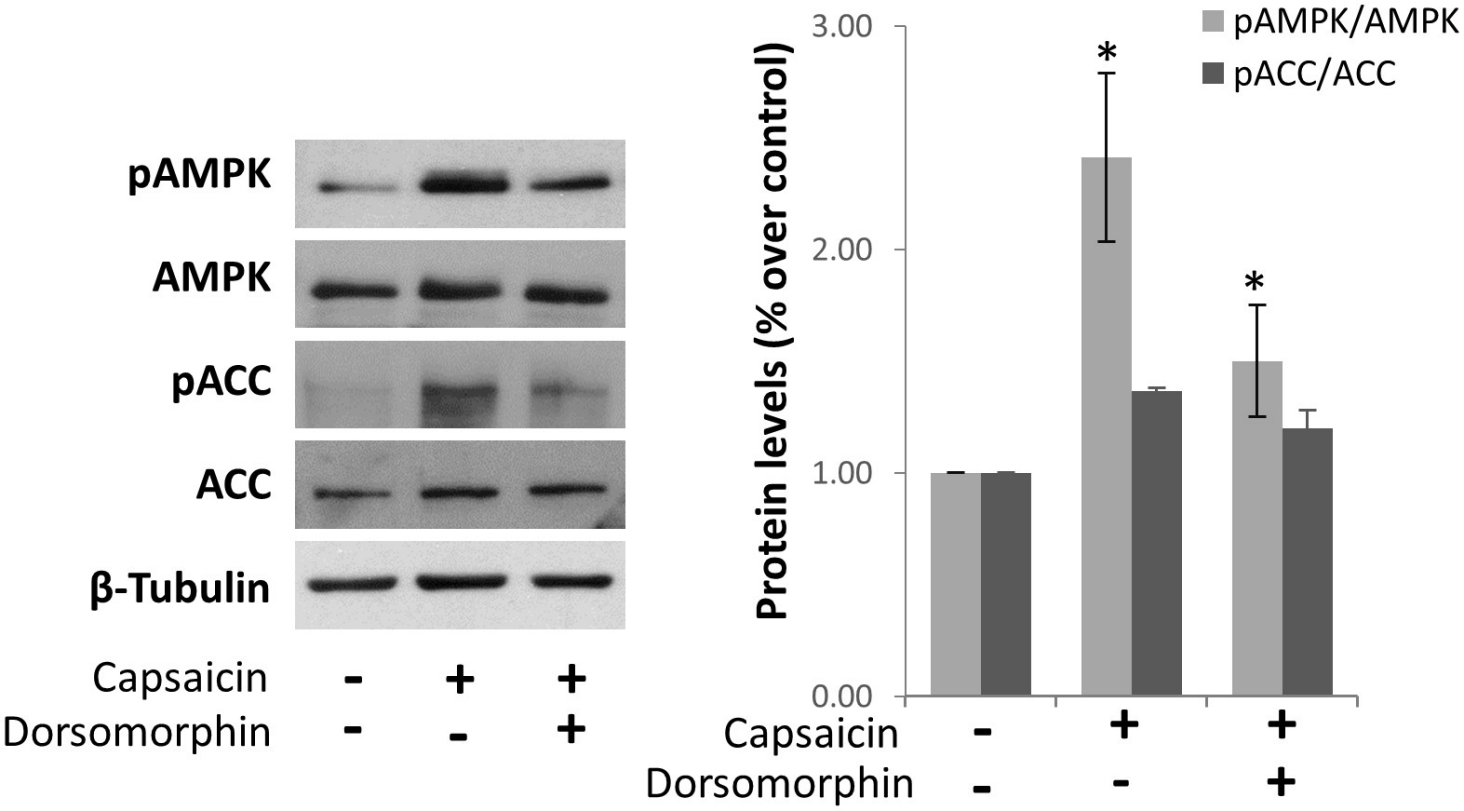
Capsaicin activates AMPK in HepG2 cells. Cells were incubated with 200μM capsaicin in the presence or not of 10 μM dorsomorphin during 1h and levels of phospho-AMPK, phospho-ACC and their corresponding total forms were determined by Western blot. β-tubulin serves as a loading control. The densitometric analysis of bands is shown on the right. Data are presented as the mean ± SEM of three different experiments. *, p<0.05 compared with the control group and # p< 0.05 compared with capsaicin by the Student’s t test.

To study whether capsaicin activates AMPK in hepatocellular carcinoma HepG2 cells, we incubated cells with capsaicin for 1 hour and determined AMPK phosphorylation in Thr172 which is within the catalytic subunit (alpha) of AMPK and a hallmark of AMPK activation, as well as the phosphorylation of the AMPK downstream target Acetyl CoA carboxylase (ACC). According to previous data from our group and others, we choose a dose of 200 μM of capsaicin which is an intermediate dose of those normally used to inhibit cell proliferation [3, 18–22]. As shown in Fig 1 and S1 Fig, the phosphorylation of both AMPK and ACC were increased after incubation with capsaicin indicating an AMPK activation. As expected, this effect was reversed with the pre-treatment with the AMPK inhibitor Dorsomorphin (also known as compound C).

### The Ca-calmodulin kinase kinase β is involved in AMPK activation by capsaicin in HepG2 cells

**Fig 2:**
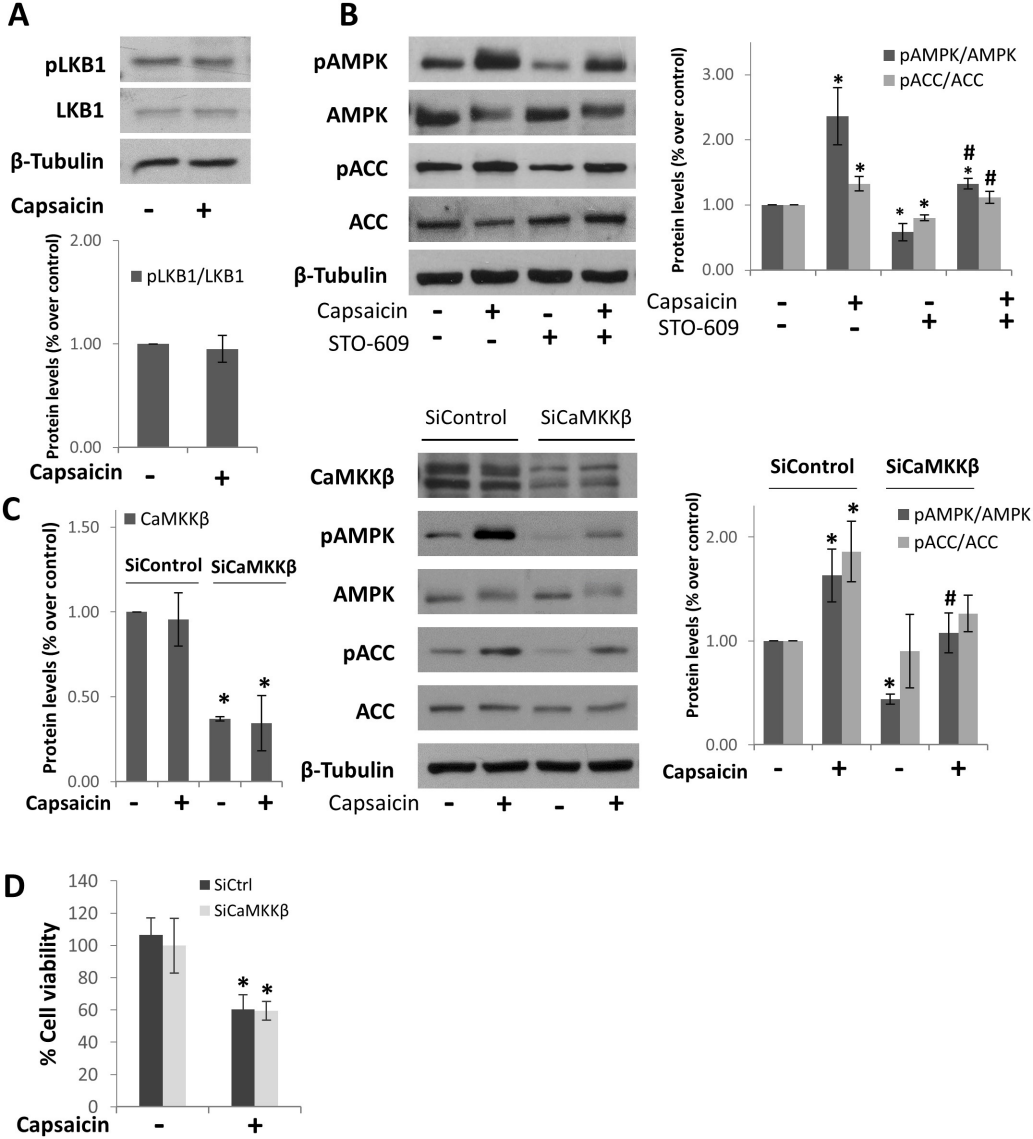
The Ca-calmodulin kinase kinase β is involved in AMPK activation by capsaicin in HepG2 cells. A, Cells were incubated with 200μM capsaicin during 1h and levels of phospho-LKB1, and total LKB1 were determined by Western blot. β-tubulin serves as a loading control. The densitometric analysis of bands is shown below. B, Effect of the CaMKK inhibitor STO-609 on AMPK activation. Cells were pre-incubated with 10 μM STO-609 and then treated with 200μM capsaicin during 1h. Levels of phospho-AMPK, phospho-ACC and their corresponding total forms were determined by Western blot. β-tubulin serves as a loading control. The densitometric analysis of bands is shown on the right. C, Effect of CaMKKβ knock down on AMPK activation. Cells were transfected with scrambled siRNA (siControl) or selective CaMKKβ siRNA and incubated with 200μM capsaicin during 1h. Levels of phospho-AMPK, phospho-ACC and their corresponding total forms were determined by Western blot. β-tubulin serves as a loading control. Left, densitometric analysis of CaMKKβ. Right, densitometric analysis of pAMPK and pACC. D, Cell viability determined by MTT of cells transfected with scrambled siRNA (siControl) or selective CaMKKβ siRNA. Data are presented as the mean ± SEM of three different experiments. *, p<0.05 compared with the control group and # p< 0.05 compared with capsaicin by the Student’s t test.

AMPK can be phosphorylated by two main kinases, LKB1 and the calcium/calmodulin-dependent protein kinase kinase β, CaMKKβ. To study which kinase was involved in the capsaicin-induced AMPK activation, we determined phosphorylation levels of LKB1 in ser428 after cell treatment with capsaicin. As shown in Fig 2A and S2 Fig, LKB1 phosphorylation was not increased, neither total LKB1 indicating that capsaicin does not activate or increase LKB1 levels in HepG2 cells. So, we investigated the involvement of CaMKKβ. For that end, we pre-treated the cells with the CaMKK inhibitor STO-609 and determined AMPK activation. Fig 2B and S3 Fig show that pre-treatment with STO-609 prevented both the phosphorylation of AMPK and the phosphorylation of ACC indicating that CaMKKβ was the underlying mechanism of capsaicin-dependent activation of AMPK. To confirm this finding, we knocked down CaMKKβ with selective siRNA. As shown in Fig 2C and S4 Fig, downregulation of CaMKKβ blocked the capsaicin-induced phosphorylation of AMPK as well as that of ACC, confirming that CaMKKβ is the upstream kinase involved in AMPK activation by capsaicin.

### AMPK activation depends on intracellular calcium and TRPV1

**Fig 3:**
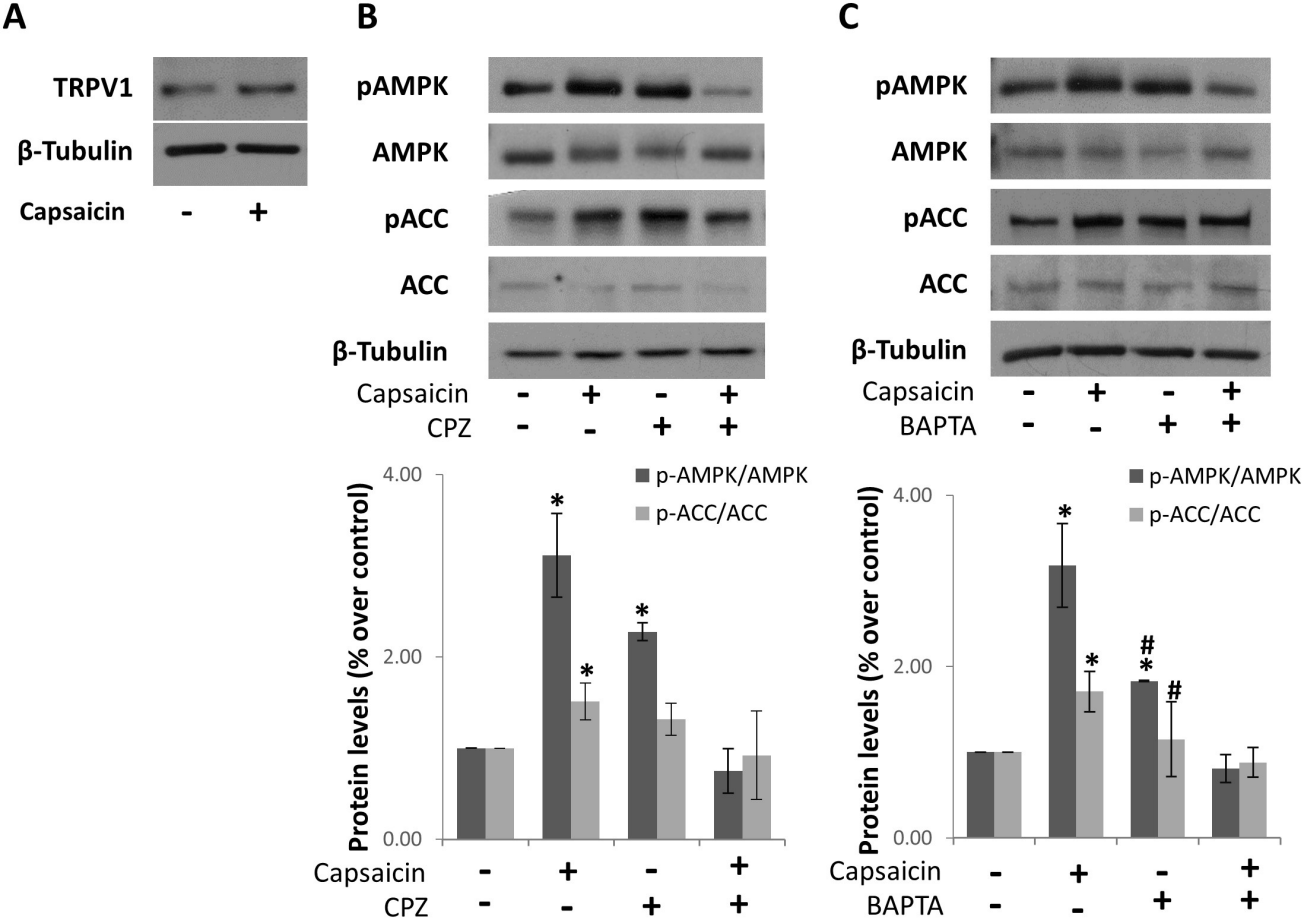
Involvement of TRPV1 and calcium in capsaicin-induced AMPK activation. A, Expression of TRPV1 receptor in HepG2 cells treated with vehicle or 200 μM capsaicn for 1h and determined by Western blot. β-tubulin serves as a loading control. B, Effect of the TRPV1 antagonist capsazepine (CPZ) on AMPK activation. Cells were pre-incubated with 10 μM CPZ and then treated with 200μM capsaicin during 1h. C, Effect of the calcium chelator BAPTA on AMPK activation. Cells were pre-incubated with 5 μM BAPTA and then treated with 200μM capsaicin during 1h. Levels of phospho-AMPK, phospho-ACC and their corresponding total forms were determined by Western blot. β-tubulin serves as a loading control. The densitometric analysis of bands is shown on the right. Data are presented as the mean ± SEM of three different experiments. *, p<0.05 compared with the control group and # p< 0.05 compared with capsaicin by the Student’s t test.

CaMKKβ is usually activated by Ca^2+^ and calmodulin [23, 24]. Capsaicin may increase intracellular calcium concentration by activating the transient potential vanilloid type receptor, TRPV1, which has been described as a capsaicin receptor. By other hand, recent data indicate that cytosolic calcium increase, causes a synergistic activation of AMPK by AMP [25]. We therefore analyzed the role of the TRPV1 receptor and intracellular calcium on AMPK activation by capsaicin. We first corroborated the expression of the TRPV1 receptor in HepG2 cells (Fig 3A and S5A Fig). We then pre-incubated the cells with the TRPV1 antagonist capsazepine (CPZ) and determined capsaicin-induced AMPK and ACC phosphorylation. As shown in Fig 3B and S5B Fig, CPZ reduced the activation of AMPK and the phosphorylation of ACC. When cells were pre-treated with the intracellular calcium chelator BAPTA, the phosphorylation of AMPK and that of ACC were totally inhibited (Fig 3C and S6 Fig). These findings indicate that capsaicin needs TRPV1 and intracellular calcium to activate AMPK in HepG2 cells.

### The antiproliferative effect of capsaicin involves AMPK activation

**Fig 4:**
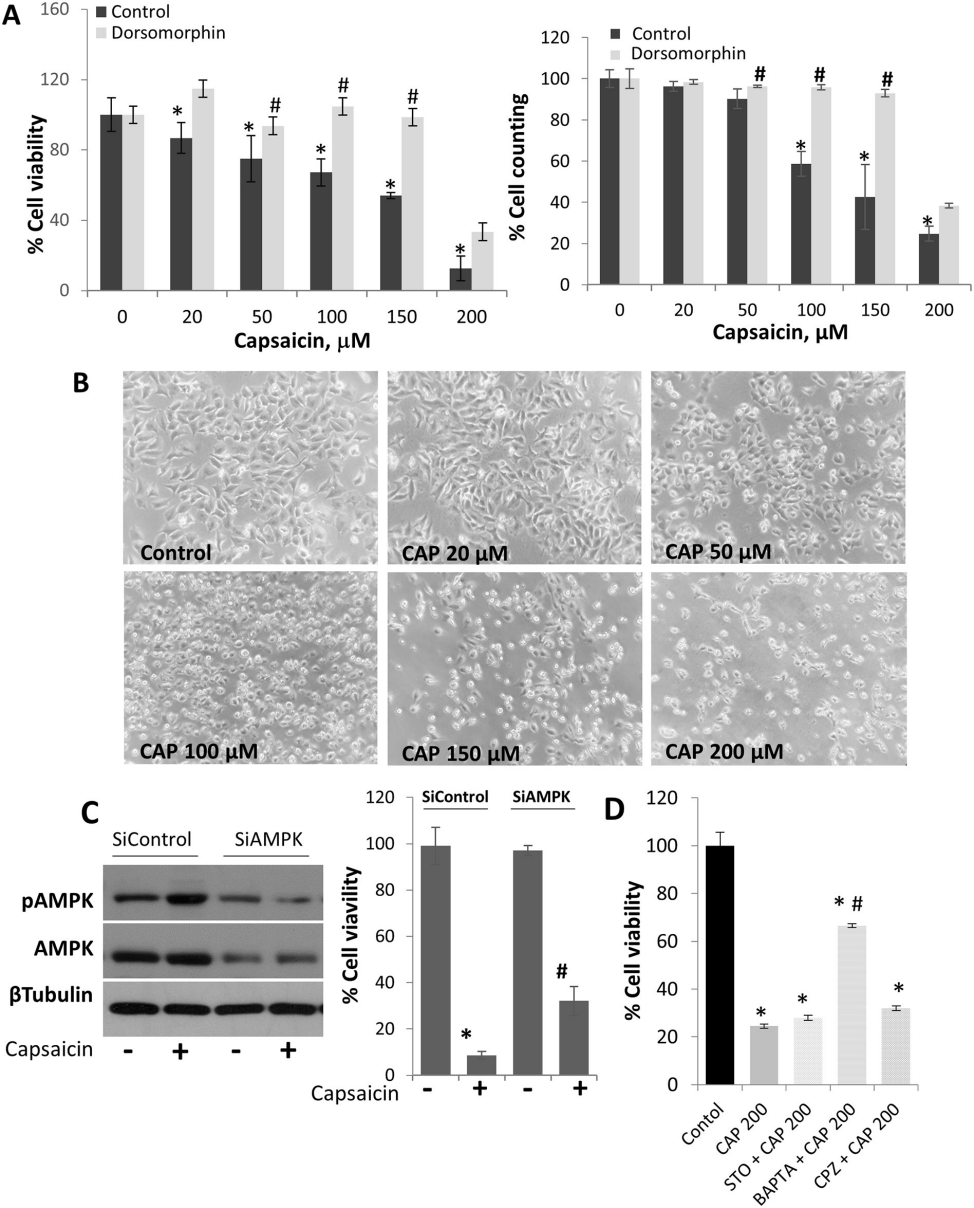
AMPK activation is involved in capsaicin-induced cell death. A, Effect of the AMPK inhibitor dorsomorphin on HepG2 cell proliferation. Cell were pre-incubated with 10 μM dorsomorphin and then treated with increasing doses of capsaicin for 24 hours. Left, cell viability determined by MTT. Right, cell counting with trypan blue. B, microphotographs of cells at the end of the treatment observed by phase contrast. C, Effect of AMPK knock-down on capsaicin-induced HepG2 cell death. Cells were transfected with scrambled siRNA (siControl) or selective AMPK siRNA and incubated with 200μM capsaicin during 24h. D, Effect of inhibitors on capsaicin-induced HepG2 cell death. Cells were pre-incubated with the different inhibitors and then incubated with 200μM capsaicin during 24h. Cell viability was determined by MTT. Data are presented as the mean ± SEM of three different experiments. *, p<0.05 compared with the control group and # p< 0.05 compared with capsaicin by the Student’s t test.

We then investigated the effect of AMPK activation on HepG2 cells proliferation. To that end, we pre-treated the cells with Dorsomorphin and with increases doses of capsaicin for 24 hours and tested cell proliferation by MTT and cell counting. As shown in Fig 4A, capsaicin dose-dependently reduced cell viability and cell proliferation. The inhibitory effect of capsaicin on cell viability was blocked by the AMPK inhibitor dorsomorphin (Fig 4A) suggesting that capsaicin inhibits cell proliferation through AMPK. Morphology of cells observed by phase contrast indicates that capsaicin induces cell shrinkage (Fig 4B). To confirm the involvement of AMPK in capsaicin-induced cell death, AMPK was knocked down by transfecting cells with selective siRNA (Fig 4B and S7 Fig). AMPK knock down reduced the antiproliferative effect of capsaicin in HepG2 cells (Fig 4B). Accordingly, pre-treatment of cells with STO-609, BAPTA or capsazepine, reduced the inhibitory effect of capsaicin, although it was statistically significant only in BAPTA-treated cells (Fig 4D).

### Capsaicin induces cell death by a mechanism involving ROS generation, autophagy and apoptosis

**Fig 5:**
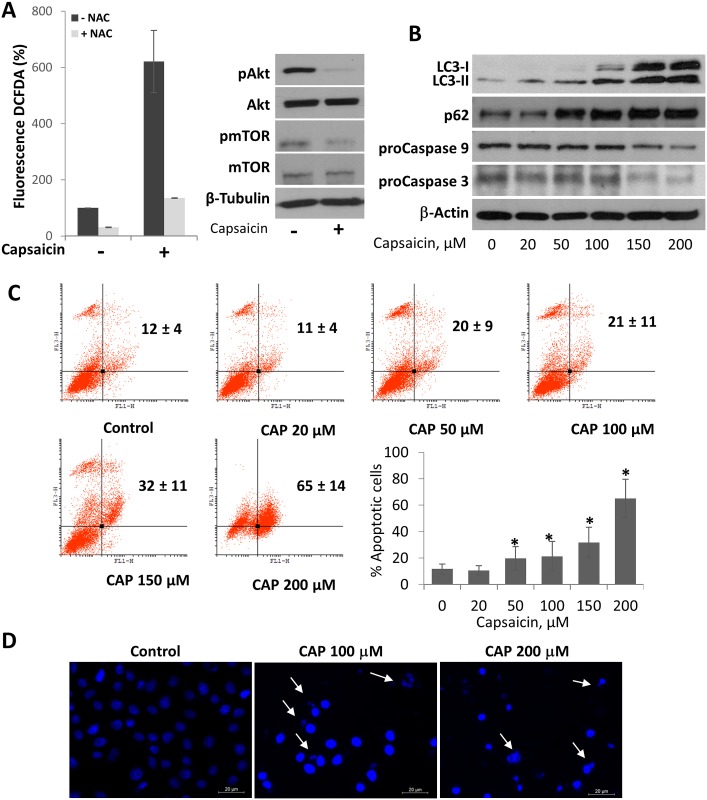
Mechanism of capsaicin-induced cell death. A, Reactive oxygen species (ROS) generation by capsaicin treatment in HepG2 cells. Cells were treated with vehicle (control) or 200 μM of capsaicin in the presence or not of the ROS scavenger N-Acetyl-l-Cysteine (NAC) for 1hand ROS were measured by flow cytometry using the dye DCFDA. B, cells were incubated with the indicated doses of capsaicin, for 24h and levels of LC3, SQSTM1/p62 procaspase-9 and procaspase-3 were determined by Western blot. β-Actin was determined as loading control. C, HepG2 cell were treated with vehicle (control) or the indicated doses of capsaicin, for 24h and then stained with Annexin V and PI. The graphs represent PI fluorescence (Y axe) versus Annexin V fluorescence (x axe). The early apoptotic cells (AnnexinV-positive, PI-negative cells) and the late apoptotic cells (Annexin V-positive, PI-positive cells) are indicated as the percentage of gated cells. Histogram represents the late apoptotic cells for each dose. Data are the mean ± SEM of three experiments. *p<0.05 vs control compared by the Student’s t test D, Cells were treated with vehicle (control) or the indicated doses of capsaicin, for 24h and DAPI-stained nuclei were detected by immunofluorescence (blue) 20X. Apoptotic bodies are indicated with an arrow.

Reactive oxygen species (ROS) are signaling regulators of several processes including apoptosis and autophagy. To investigate whether treatment with capsaicin induced ROS generation in HepG2 cells, intracellular ROS were determined by the probe DCFDA. As shown in Fig 5A, treatment of HepG2 cells with capsaicin induced a notably increase in the intracellular levels of ROS which was abrogated by the ROS scavenger *N*-Acetyl-l-cysteine (NAC).

To further explore the underpinning mechanism of capsaicin-induced cell death we analyzed the Akt/mTOR pathway which regulates proliferation in many cell types. Treatment of cell with capsaicin caused a strong inhibition of Akt phosphorylation at ser-473 as well as a decrease of mTOR phosphorylation at ser-2448 (Fig 5A and S8 Fig). In many cells and systems, AKT/mTOR signaling pathway inhibition has been related with autophagy induction. We then investigated whether capsaicin promoted autophagy in HepG2 cells by determining the levels of the LC3-II isoform which results of LC3 lipidation and is a hallmark of autophagy. As can be observed in Fig 5B and S9 Fig, capsaicin induced a dose-dependently increase of both LC3-I (nonlipidated) and LC3-II (lipidated) forms. The LC3 proteins are involved in phagophore formation and, as autophagy is a dynamic process, LC3 accumulation may be indicative of either increased autophagosome formation, or reduced autophagosomes turnover [26]. To discriminate between this two conditions, we determined levels of SQSTM1/pP62. SQSTM1/pP62 protein is defined as a cargo receptor for selective autophagy serving as a link between LC3 and ubiquitinated substrates. Inhibition of autophagy correlates with increased levels of SQSTM1 [21]. Treatment of HepG2 cells with capsaicin produced an increase in SQSTM1/pP62 levels (Fig 5B), pointing to an autophagy blockage. Such situation correlated with a reduction of procaspase 9 and procaspase 3 which is indicative of apoptosis (Fig 5B). These results indicate that capsaicin induces autophagy flux obstruction which probably leads to cell death by apoptosis. To corroborate this notion, we analyzed apoptosis in capsaicin-treated cells by anexin V/PI labelling and flow cytometry. As seen in Fig 5C, treatment with capsaicin induced a dose-dependent increase in apoptotic cells, confirming that capsaicin induces hepatocarcinoma HepG2 cell death by promoting apoptosis. Apoptotic bodies and nuclei shrinkage could be observed by fluorescence microscopy of cells stained DAPI probe which specifically stains nuclei (Fig 5C).

## Discussion

In the past decade’s phytochemicals have gain attention as chemotherapeutic agents because of their inhibition of critical tumor properties such as growth, invasion, progression and metastasis [1]. In addition, phytochemicals have low toxicity, low cost, and public acceptance as dietary supplements. In particular capsaicin has demonstrated to exert anticancer effects in a variety of tumor types including hepatocarcinoma although the underlying mechanism remains unclear. Additionally, experimental end epidemiological data also demonstrate that capsaicin exerts anti-obesity properties and increase energy expenditure by activation of catabolic routes, thus providing a novel strategy to modulate cancer cell metabolism. Metabolic cell homeostasis is governed by the key energy sensor AMPK. In fact, targeting AMPK has become a novel strategy for cancer prevention and treatment [27]. Thus, in this study, we investigated the mechanism underpinning AMPK activation by capsaicin and the involvement of AMPK in capsaicin-induced cell death in hepatocarcinoma cells HepG2. We found that capsaicin activates AMPK by inducing its phosphorylation by CaMKKβ. We have recently demonstrated that capsaicin sensitized HCC cells to sorafenib by activation of AMPK [28] which is in line with results found in this study. Recent data by Zang et al. demonstrated that the treatment of HepG2 cells with the non-pungent capsaicinoid, capsiate, increased AMPK and ACC phosphorylation and impact lipid metabolism [29]. In bladder cancer cells capsaicin at 300 μM increase AMPK phosphorylation which authors relate with an autophagy induction by ROS, which is in good agreement with our results [30]. Nevertheless, although our knowledge this is the first study that analyzes the mechanism involved. We have found that capsaicin-induced AMPK activation depends on TRPV1 receptor, intracellular calcium and CaMKKβ, which become potential targets to modulate AMPK activity and cell death.

We have also found that capsaicin increased intracellular ROS generation, inhibited Akt/mTOR produced an autophagy blockage and induced apoptosis, although the involvement of AMPK in this processes remains to be fully elucidated. The role of AMPK in autophagy is complex and highly dependent on both cell type and metabolic conditions. In liver cells, AMPK suppresses autophagy at the level of cargo sequestration, which is in good agreement with our results, whereas it appears to stimulate autophagy in many other cell types, including fibroblasts, colon carcinoma cells and skeletal muscle [21]. Results by Samari et al., demonstrated that AMPK activation with AICAR suppressed autophagy in liver cells [31], which is in line with our results. We have observed an increase in ROS and inhibition of the Akt/mTOR signaling pathway in HCC cells treated with capsaicin. Previous data demonstrated that ROS are essential for autophagosome formation by a mechanism partially dependent on Akt inhibition which in turn inhibits mTOR and activates autophagy [32]. By other hand, AMPK activates autophagy by inhibition of mTOR through the direct phosphorylation of Raptor, [21]. In addition to regulation of mTORC1 activities, AMPK can directly phosphorylate ULK1 to regulate mitophagy and mitochondrial homeostasis [33]. Therefore, both AMPK and Akt have vital roles in mTOR signaling pathway. It is possible that capsaicin exerts a dual role in liver autophagy, by one hand increasing LC3-II conversion and thus increasing the formation of autophagosomes, and by other hand inducing SQSTM1/p62 accumulation by blocking autophagic degradation in concordance with previous results in prostate cells [22]. This notion needs to be elucidated in future research. Our results demonstrate that capsaicin blocks the autophagy flux and induces apoptosis which correlates with Akt/mTOR inhibition and AMPK activation, although the exact contribution of both signaling pathways to capsaicin-induced cell death has not been elucidated.

These results are in good agreement with previous results by our group showing an autophagy inhibition by capsaicin in prostate cells [22]. Similar results were obtained by Lui et al. in glioma cells or by Wang et al. in osteosarcoma cells, in which capsaicin increased levels of LC3-II and of p62 protein [18, 21]. In line with this, Chen et al. found that pharmacological or genetic inhibition of autophagy further sensitized HepG2 cells to capsaicin-induced apoptosis [19]. Similarly, the inhibition of autophagy, by using the specific inhibitor bafilomycin A or Beclin 1 knock-down enhanced the capsaicin-induced cell death in bladder cancer cells [30]. Authors also observed an increase in LC3-II and SQSTM1/p62 in capsaicin-treated bladder cancer cells [30]. However, other authors observed a SQSTM1/p62 decrease after capsaicin treatment but doses used were higher and time longer than ours [20].

Altogether, our data demonstrate that capsaicin activates AMPK in Hepatocellular carcinoma HepG2 cells by a mechanism involving TRPV1-calcium and CaMKKβ. AMPK activation by capsaicin reduces cell viability by a mechanism that involves ROS generation, Akt/mTOR inhibition, autophagy blockage and apoptosis. This highlights the importance to impact cell metabolism to inhibit proliferation and points to AMPK as a new therapeutic option for HCC. The activation of AMPK by capsaicin provides a new molecular mechanism for capsaicin action and contributes to the future applications of capsaicin in medicine.

## Supporting information

S1 Fig Western blot of pAMPK, AMPK, pACC and ACC in HepG2 cells treated with capsaicin. (TIF)

S2 Fig Western blot of pLKB1, and LKB1 in HepG2 cells treated with capsaicin. (TIF)

S3 Fig Western blot of pAMPK, AMPK, pACC, ACC in HepG2 cells treated with capsaicin and STO-609. (TIF)

S4 Fig Western blot of pAMPK, AMPK, pACC, ACC in HepG2 cells treated with capsaicin and in which CAMKKα or CaMKKβ were knocked-down. (TIF)

S5 Fig A, Western blot of TRPV1 in HepG2 cells treated or not with capsaicin. B, Western blot of pAMPK, AMPK, pACC and ACC in HepG2 cells treated with capsaicin, capsaicin + BAPTA and capsaicin + capsazepine.
(TIF)

S6 Fig Western blot of pAMPK, AMPK, pACC and ACC in HepG2 cells treated with capsaicin and capsaicin + BAPTA. (TIF)

S7 Fig Western blot of pAMPK and AMPK in HepG2 cells with AMPK knocked-down and treated with capsaicin. (TIF)

S8 Fig Western blot of pAkt, Akt, pmTOR and mTOR in HepG2 cells treated with capsaicin. (TIF)

S9 Fig Western blot of LC3, p62, procaspase 9 and procaspase 3 in HepG2 cells treated with capsaicin. (TIF)
