# Supplementary figures and images for: The red pepper’s spicy ingredient capsaicin activates AMPK in HepG2 cells through CaMKKβ
Source: PLoS One. 2019 Jan 29;14(1):e0211420. doi: 10.1371/journal.pone.0211420 (PMC6350977; doi:10.1371/journal.pone.0211420)

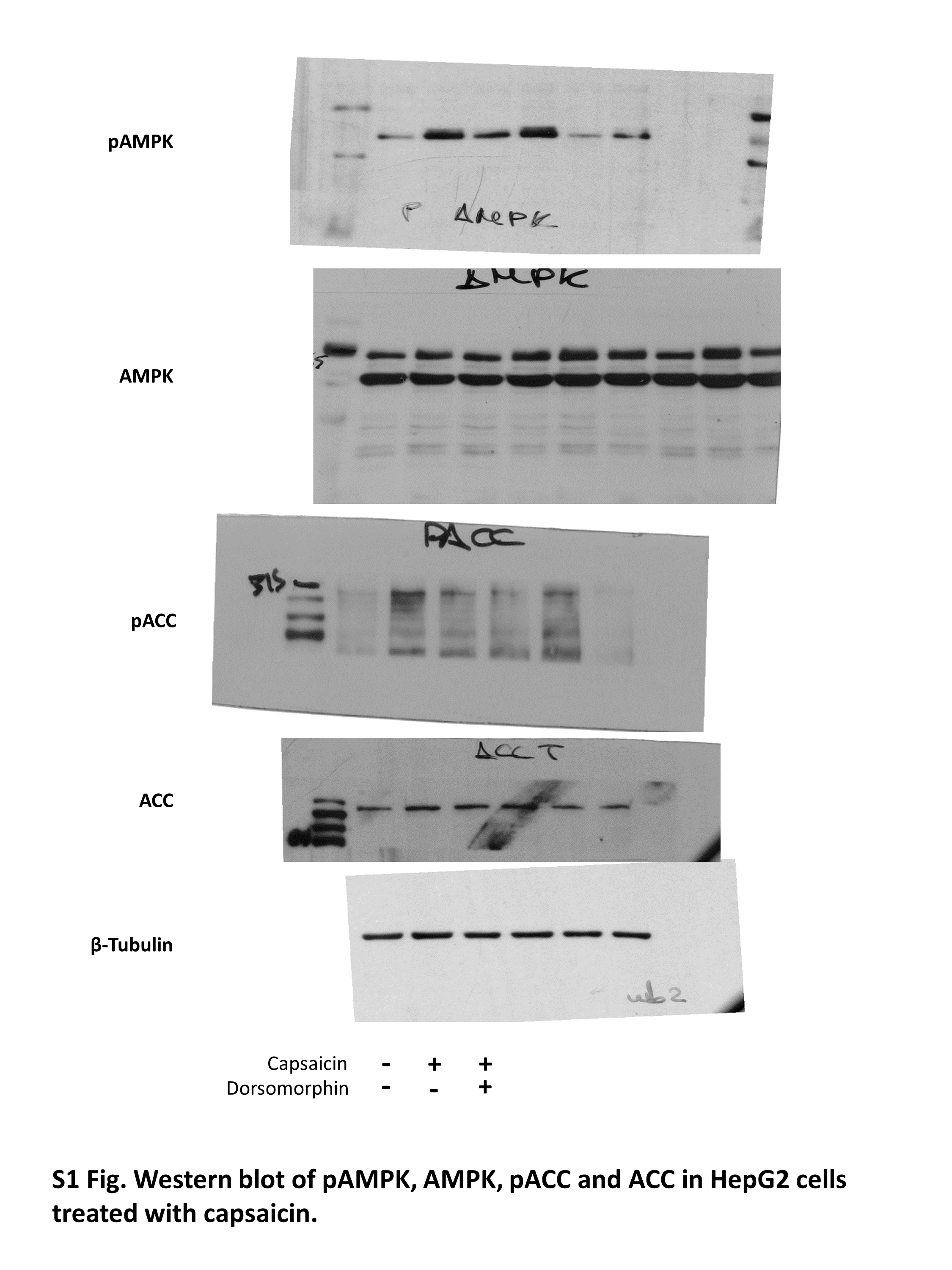

Supplement: S1 Fig — (TIF) [file pone.0211420.s001.tif]

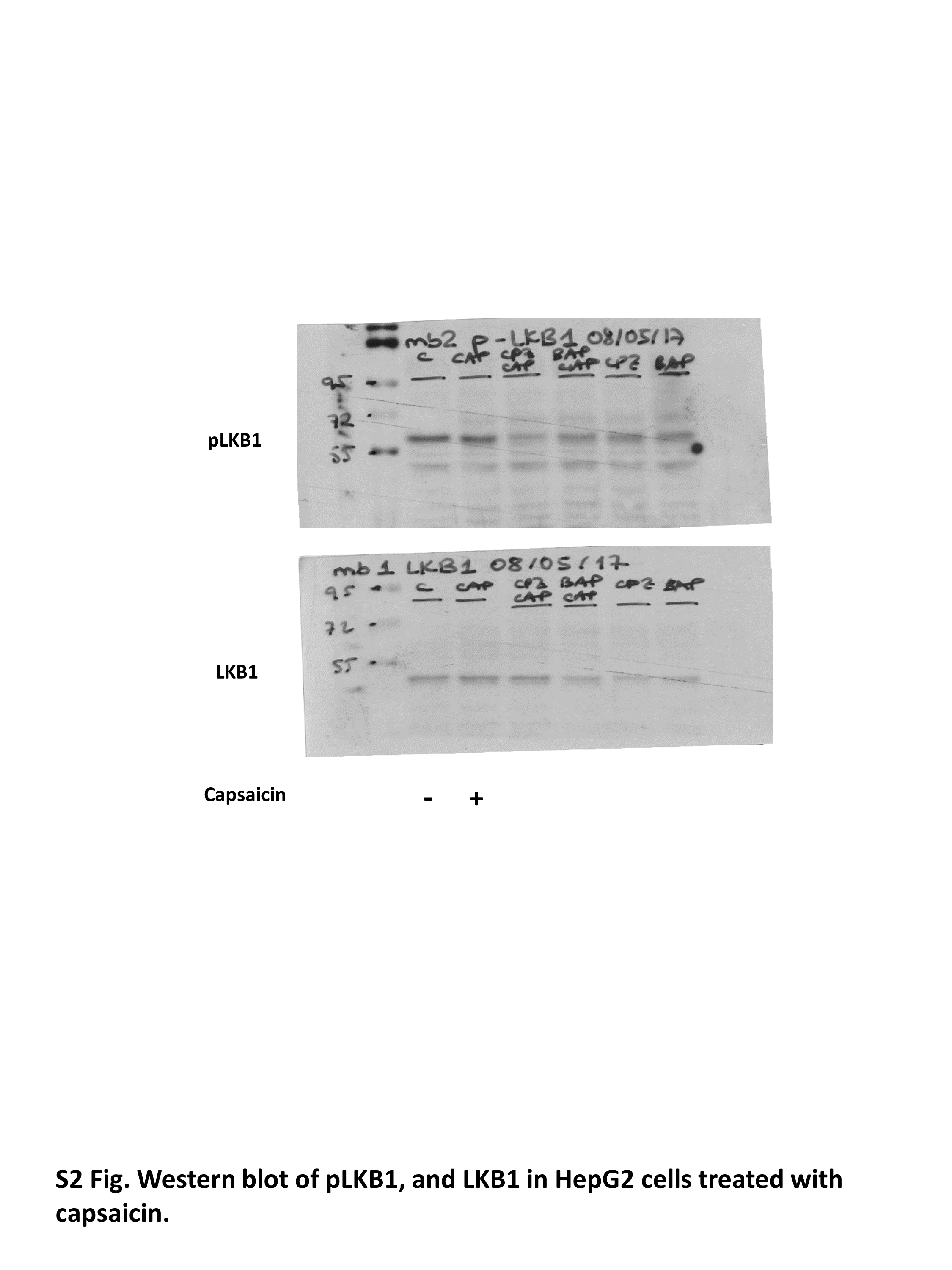

Supplement: S2 Fig — (TIF) [file pone.0211420.s002.tif]

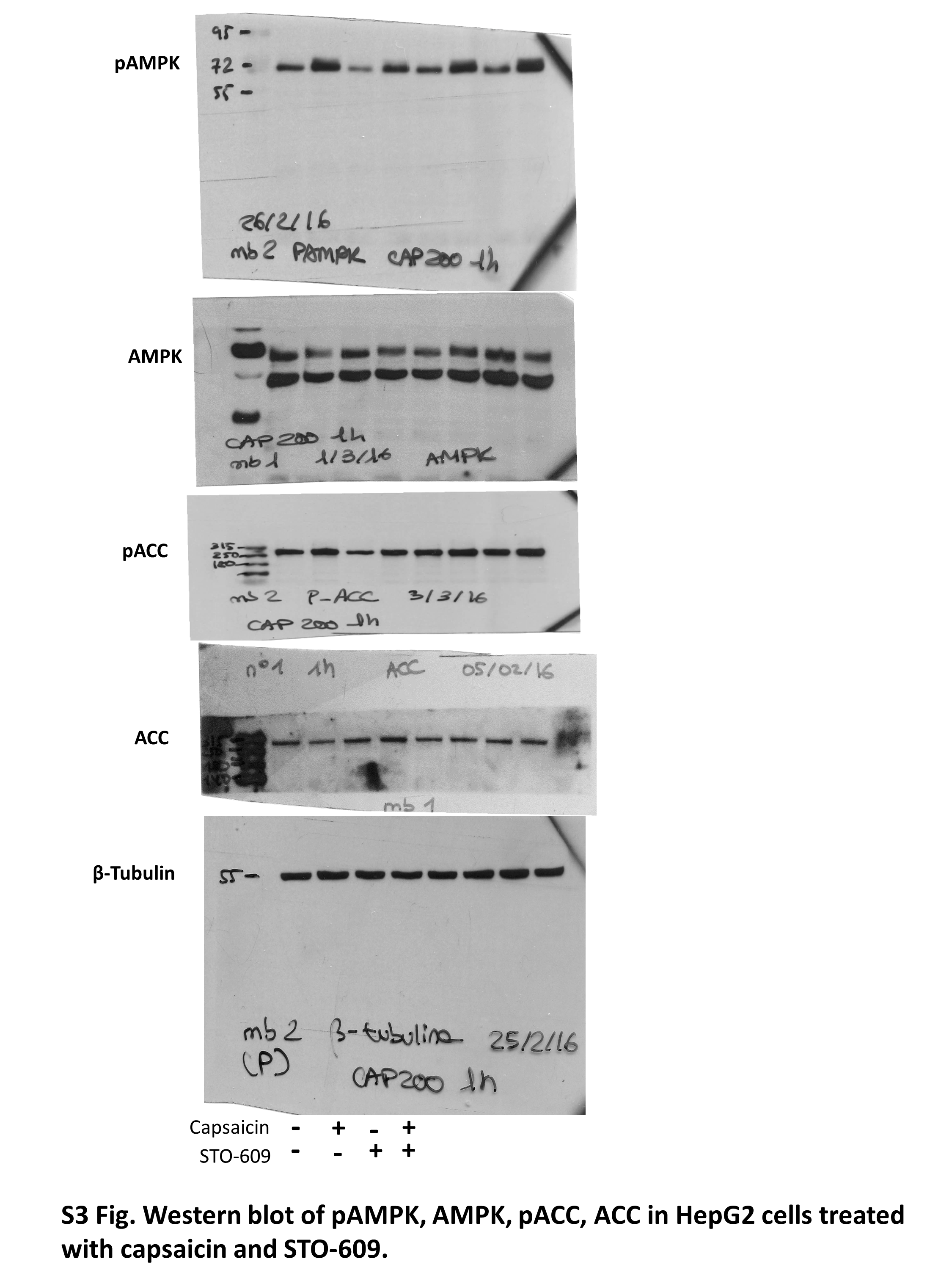

Supplement: S3 Fig — (TIF) [file pone.0211420.s003.tif]

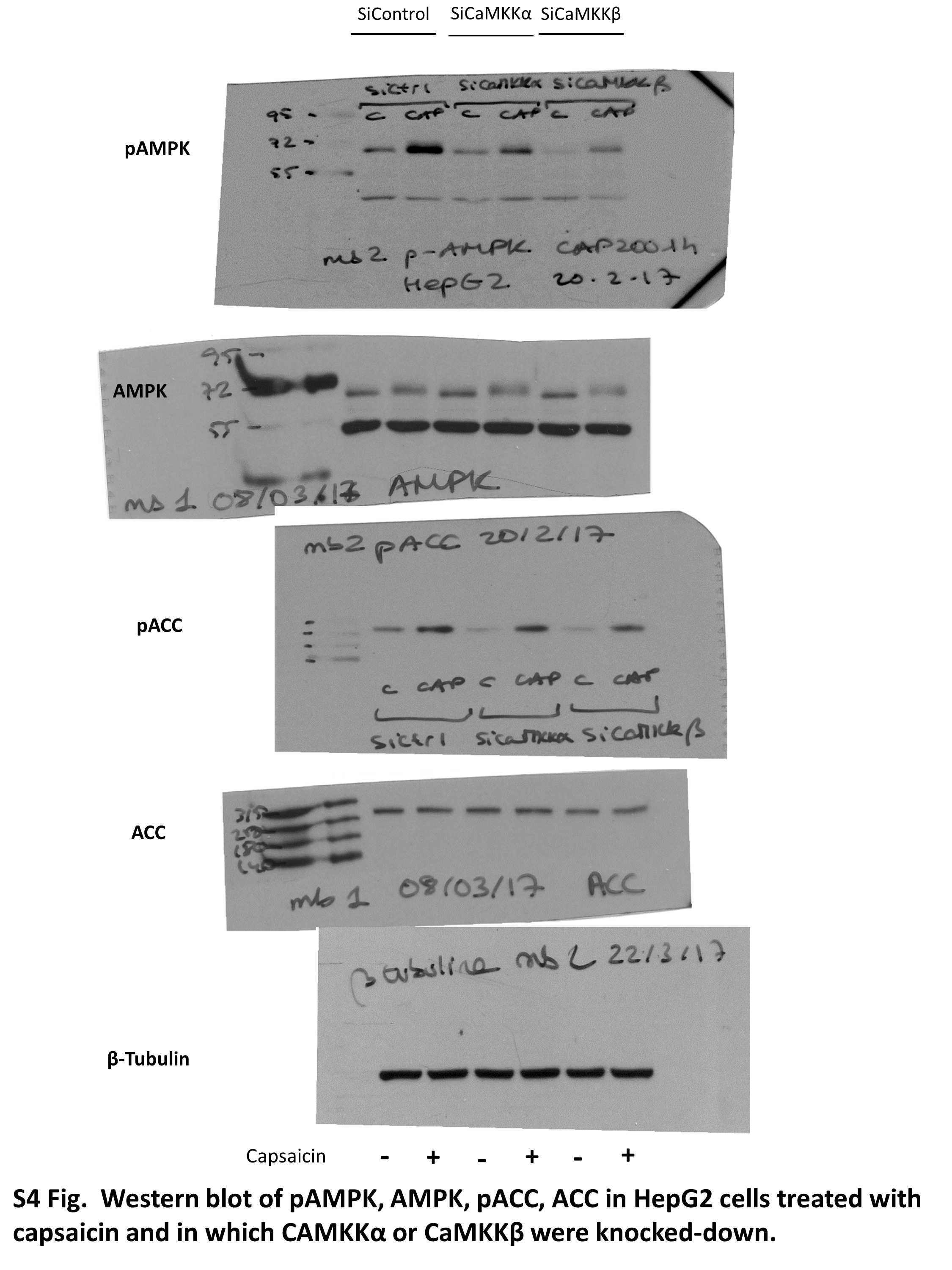

Supplement: S4 Fig — (TIF) [file pone.0211420.s004.tif]

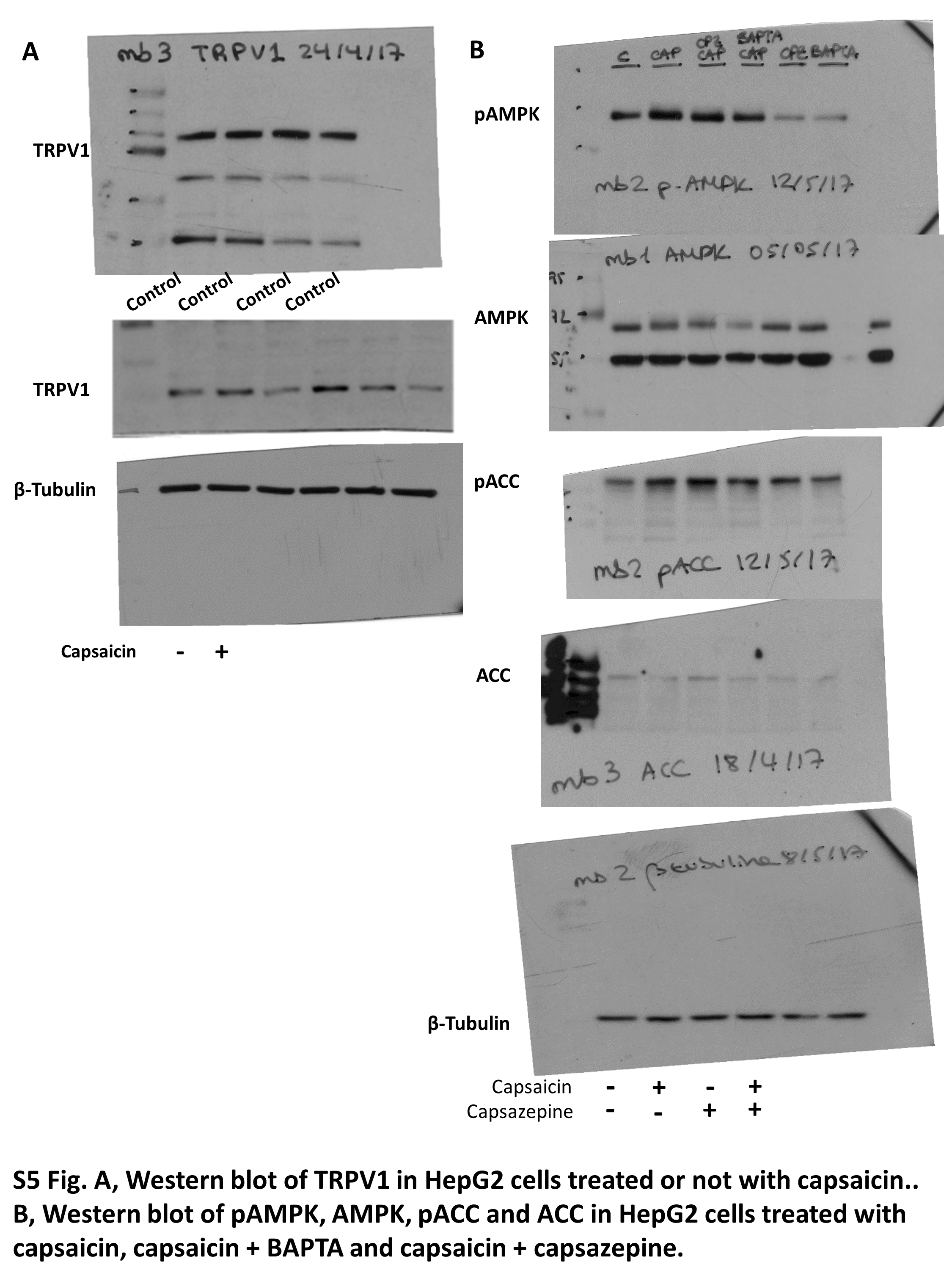

Supplement: S5 Fig — A, Western blot of TRPV1 in HepG2 cells treated or not with capsaicin. B, Western blot of pAMPK, AMPK, pACC and ACC in HepG2 cells treated with capsaicin, capsaicin + BAPTA and capsaicin + capsazepine. (TIF) [file pone.0211420.s005.tif]

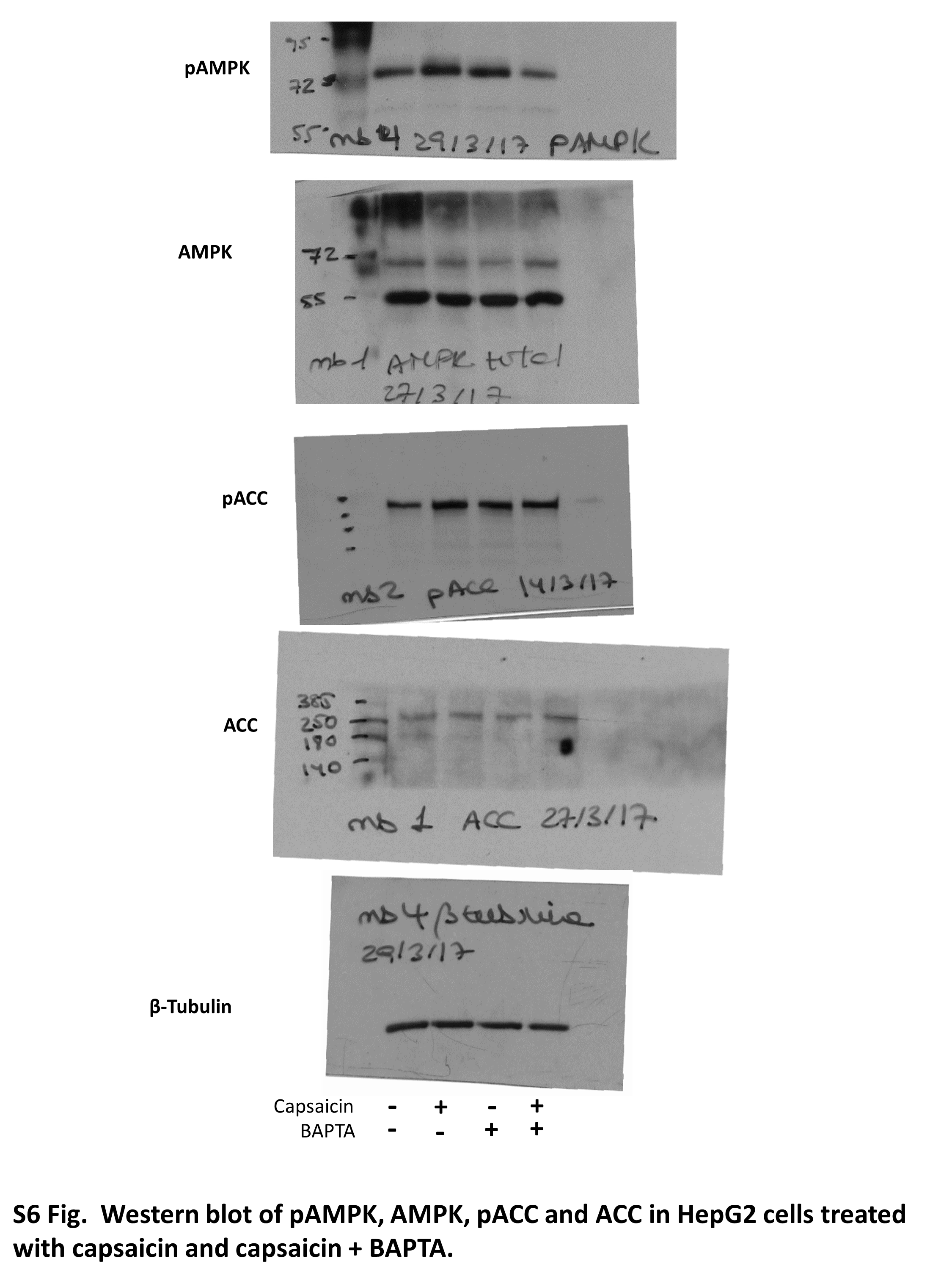

Supplement: S6 Fig — (TIF) [file pone.0211420.s006.tif]

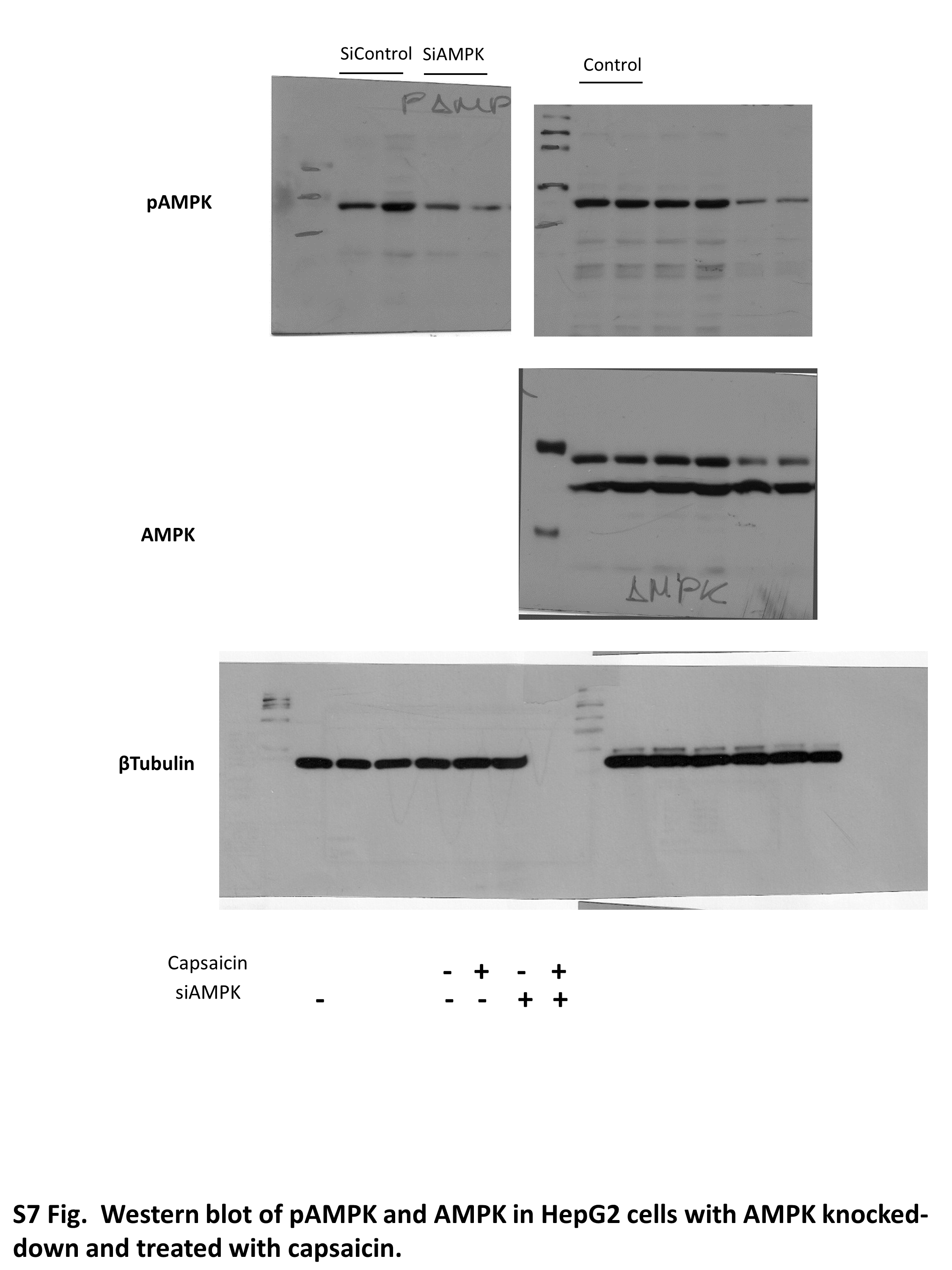

Supplement: S7 Fig — (TIF) [file pone.0211420.s007.tif]

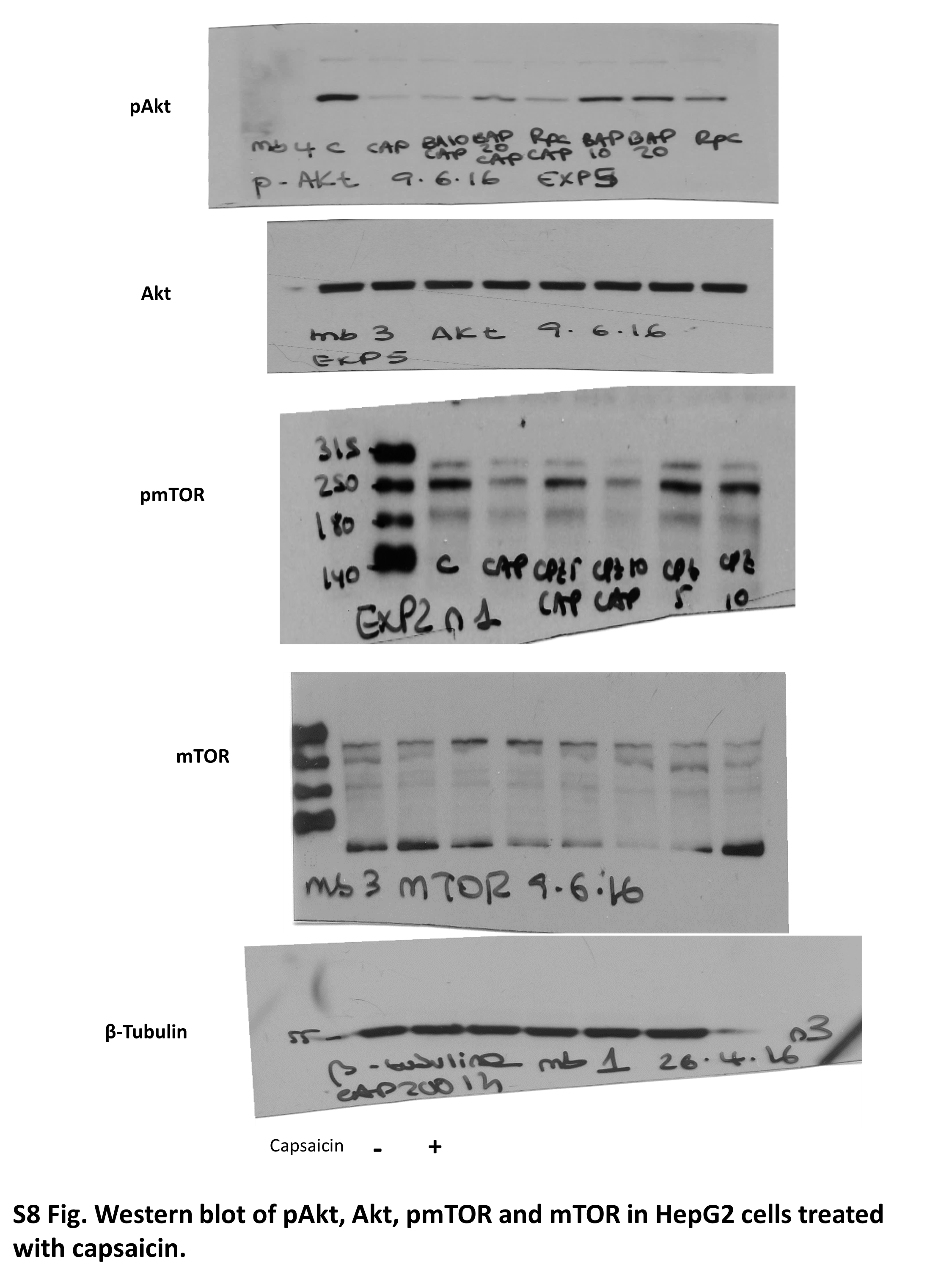

Supplement: S8 Fig — (TIF) [file pone.0211420.s008.tif]

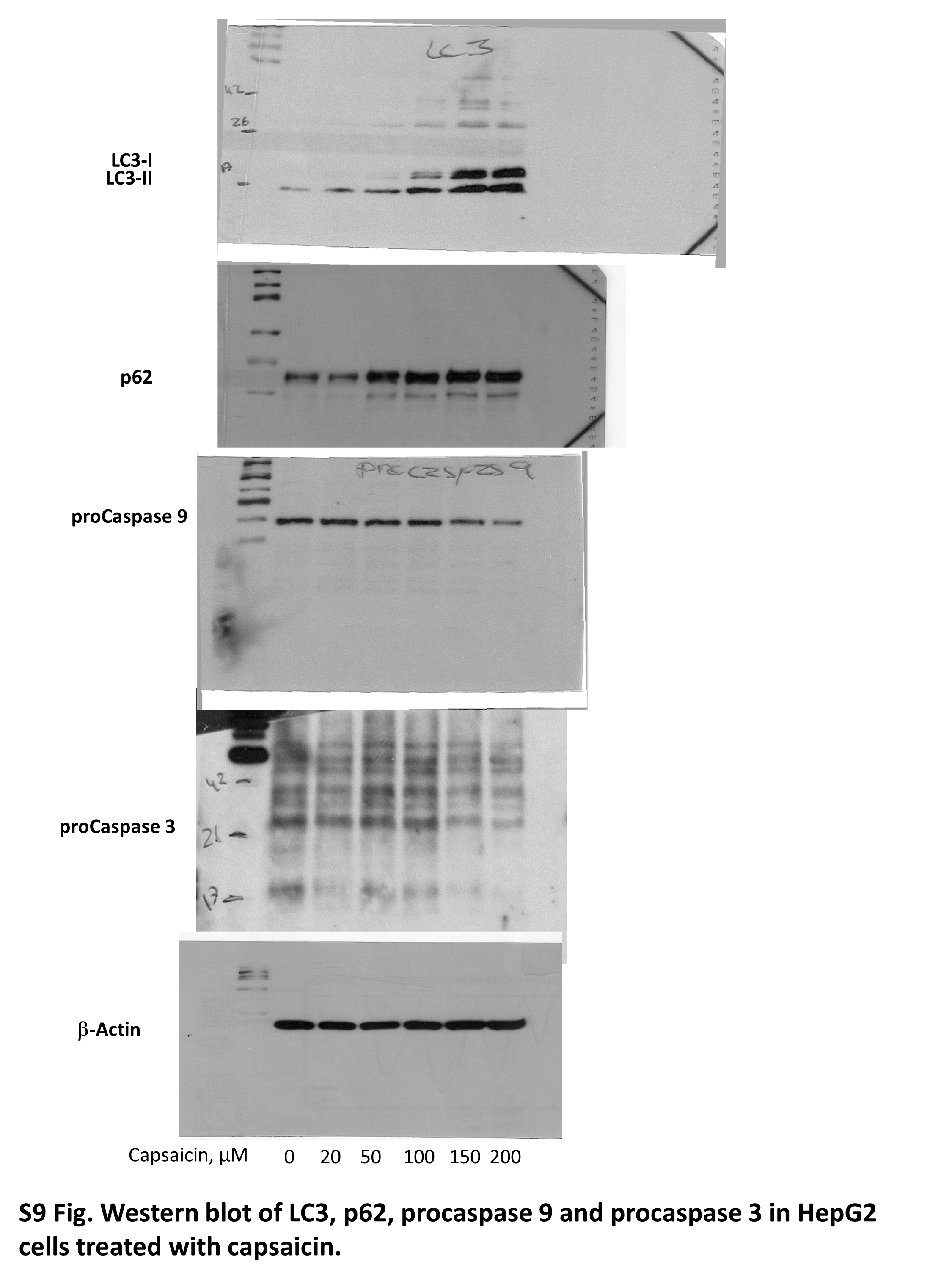

Supplement: S9 Fig — (TIF) [file pone.0211420.s009.tif]
